# Assessment of hypothalamic-pituitary-adrenal axis impairment and effects of hydrocortisone treatment in adults with Prader-Willi syndrome

**DOI:** 10.3389/fendo.2025.1517334

**Published:** 2025-06-04

**Authors:** Magdalena Góralska, Agata Pokrzywa, Agnieszka Stańczyk, Maria Libura, Tomasz Bednarczuk

**Affiliations:** ^1^ Department of Internal Medicine and Endocrinology, Medical University of Warsaw, Warsaw, Poland; ^2^ Department of Internal Medicine and Endocrinology, University Clinical Center of the Medical University of Warsaw, Warsaw, Poland; ^3^ Division of Public Health & Social Medicine, Medical University of Gdańsk, Gdańsk, Poland

**Keywords:** Prader-Willi syndrome, hypothalamic-pituitary-adrenal axis impairment, adrenal insufficiency, rare disease, hydrocortisone treatment, high dose short synacthen test

## Abstract

**Objective:**

The prevalence of hypothalamic-pituitary-adrenal impairment (HPAI) in adults with Prader Willi Syndrome (PWS) remains unclear despite its clinical relevance. The aim of our study was to assess the prevalence of HPA axis impairment in adults with PWS based on the results of the high dose short synacthen test (HDSST), as well as to analyze the effects of hydrocortisone (HCT) therapy in this population.

**Design:**

Retrospective analysis.

**Patients:**

Thirty adult patients (14 men, 16 women, aged 18–28 years) with genetically confirmed PWS. Twenty-two patients (73.3%) had been adequately treated with human recombinant growth hormone (rhGH). Due to hypogonadotropic hypogonadism, all patients received hormone replacement therapy

**Measurements:**

Physical examination included measuring height, weight and body fat percentage (using the electrical bioimpedance method). Based on HDSST results, patients were divided into two groups: with HPA axis impairment (cortisol < 500 nmol/L at 30^th^ minute), and AS (adrenal sufficiency; cortisol ≥ 500 nmol/L at the 30^th^ minute). Clinical symptoms of adrenal insufficiency (AI), body weight and body fat percentage were evaluated at baseline, after 6 and 12 months of follow-up.

**Results:**

Fourteen of the 30 patients (46.7%) showed a 30-min cortisol peak <500 nmol/L, and were assigned to the HPAI group. Peak cortisol levels at 30’ and 60’ were significantly lower in the HPAI group compared to the Control one, respectively (*P*<0.001) Correlation analysis revealed that basal cortisol was positively correlated with cortisol levels at both 30’ and 60’ of the HDSST (r = 0.872, *P* < 0.001 and r = 0.829, *P* < 0.001, respectively). Fatigue, myalgia and muscle weakness occurred more often in the HPAI group than in the Control group (90.9% vs. 20%, *P*= 0.01, 90.9% vs. 0%, *P*=0.001, respectively). All symptomatic patients with HPAI received HCT treatment (10 mg/day) in two divided doses. Fatigue, myalgiaand muscle weakness improved significantly after 12 months of HCT therapy (P<0.001). No adverse effects of HCT treatment were observed, such as weight gain, body fat percentage increase or metabolic abnormalities.

**Conclusions:**

The results of our study suggest that the HPA axis should be routinely evaluated in adult patients with PWS. Short term, low-dose HCT treatment in symptomatic patients with HPAI is safe and can reduce symptoms of fatigue, myalgia and muscle weakness. However, the benefits and adverse effects of HCT treatment in this population require confirmation in prospective, placebo-controlled randomized clinical studies.

## Introduction

Hypothalamic-pituitary disorders observed in patients with Prader-Willi syndrome (PWS) are responsible for a variety of symptoms, including severe hyperphagia and endocrine disorders (1, 2). A less understood aspect of PWS is the function of the hypothalamic-pituitary-adrenal (HPA) axis (3). It has previously been hypothesized that undiagnosed adrenal insufficiency might lead to an increased risk of a sudden death in PWS, particularly during episodes of stress and infections (4–6), given the increased mortality rate this population: 3% per year across all ages and 7% per year in those over 30 years of age (5, 6). The following clinical findings seem to suggest that HPA axis impairment (HPAI) may indeed be a part of the clinical picture of PWS: (i) the presence of a hypothalamic dysfunction with multiple hormone deficiencies (2), consistent with pituitary hypoplasia in brain imaging (7, 8), (ii) the autopsy findings of hypoplastic adrenal glands in PWS patients dying suddenly, and (iii) reports of sudden death cases with a documented poor cortisol response after an ACTH test (9, 10). However, the prevalence of HPAI in PWS, as assessed with laboratory tests, varies from 0% to 60%. Such significant discrepancies appear to be related to (i) the test used: -methyrapon test, insulin tolerance test (ITT), low-dose short Synacthen test (LDSST) or high-dose short Synacthen test (HDSST), (ii) the accepted cut-off points of cortisol used to diagnose central adrenal insufficiency (CAI) and (iii) the age of patients with PWS (some studies suggest a progressive HPA dysfunction throughout life) (2, 11–14). A further complication stems from the fact that the usual clinical signs and symptoms of adrenal insufficiency are difficult to assess in PWS patients; for instance, myalgia muscle weakness and fatigue are typical of PWS, whereas loss of appetite and weight loss are normally not observed in individuals with this syndrome (15, 16) (2). Given the possible side effects (such as weight gain and metabolic complications), the hydrocortisone (HCT) therapy in PWS patients with HPAI has so far been approached with great caution among clinicians. At present, some authors recommend stress doses of hydrocortisone in patients with suspected HPA disfunction, while others recommend close observation and monitoring (2, 11–14).

The aim of this study was to assess the prevalence of HPA impairment in a single center cohort of adults with PWS using HDSST and, subsequently, to analyze the effects of HCT therapy in symptomatic patients with HPAI.

## Materials and methods

### Patients

Thirty consecutive patients with PWS (14 men, 16 women, aged 18–28 years), referred to the Department of Endocrinology Medical University of Warsaw in the years 2016-2021, were included in the study. All patients had been previously treated in the Departments of Pediatric Endocrinology and Diabetes. The diagnosis of PWS was based on the clinical phenotype and genetic testing.

None of the patients had been treated with systemic or topical glucocorticosteroids (GCS) for the last six months. Twenty-two patients (73.3%; 12 women and 10 men) had been treated with recombinant human growth hormone (rhGH) in the last 12 months. GH therapy was continued in all previously qualified subjects. The IGF-1 levels were age- and gender-appropriate at baseline and follow-up. Due to hypogonadotropic hypogonadism, all patients received hormone replacement therapy (estradiol and progesterone in females or testosterone in males). Moreover, 16/30 (53.3%) patients received LT-4 supplementation. Dyslipidemia was identified in 14 out of 30 patients (46.7%) and was managed with dietary interventions; no statins were prescribed. All patients had normal vitamin D levels while on supplementation. None of the patients had anemia or iron deficiency.

Physical examination included measuring height, weight and body fat percentage in fasting conditions. Standing height was determined by a stadiometer WPL150. Body weight (to nearest 0.1kg), and body fat percentage were measured using the electrical bioimpedance method with Tanita Body Composition Analyser Type TBF- 300 MA (17, 18). Body Mass Index (BMI) was defined as weight (kg)/height (m^2^). Following the World Health Organization criteria, the BMI cut off points of 18.5-25.0 to define normal weight, 25.0-30.0 to define overweight and >30.0 to define obesity, were used. Physical examination also included determination of arterial pressure (including orthostatic hypotension) and heart rate.

Any concomitant diseases and additional treatments were recorded (**Table 1**).

**Table 1:** Clinical characteristics of studied patients with Prader-Willi syndrome at I VISIT.

Variable	Total (n=30)	HPAI group (n=14)	Control group (n=16)	*P* value (HPAI vs. Control)
Age, y	19.5 (18.0-28.0)	20.0 (18.0-26.0)	18.0 (18.0-28.0)	0.16^a^
Sex Male Female	14 (46.7) 16 (53.3)	8 (57.1) 6 (42.9)	6 (37.5) 10 (62.5)	0.28^b^
Genetic abnormality UPD15 15q (11-13) deletion	7 (23.3) 23 (76.7)	3 (21.4) 11 (78.6)	4 (25.0) 12 (75.0)	0.58^c^
Weight, kg	72.3 (42.0-168)	68.0 (42.0-120)	77.0 (48.0-168)	0.39^a^
BMI, kg/m^2^	24.9 (18.0-48.5)	24.4 (18.8-44.0)	26.1 (18.0-48.5)	0.74^a^
Obesity	10 (33.3)	3 (21.4)	7 (43.8)	0.18^c^
Body Fat, %	26.5 (12.8-64.0)	23.8 (16.0-64.0)	33.4 (12.8-55.0)	0.45 ^a^
Concomitant diseases Hypothyroidism Hypogonadism IFG Lipid disorders Mental retardation Mild Moderate/severe	16 (53.3) 30 (100) 14 (46.7) 14 (46.7) 7 (23.3) 23 (76.7)	7 (50.0) 14 (100) 7 (50.0) 5 (35.7) 4 (28.6) 10 (71.4)	9 (56.3) 16 (100) 7 (43.8) 9 (56.3) 3 (18.8) 13 (81.3)	0.73^b^ NA^b^ 0.73^b^ 0.22^c^ 0.42^c^
Current treatment Growth hormone Levothyroxine HRT Vitamin D Psychiatric ^d^	22 (73.3) 16 (53.3) 30 (100) 30 (100) 15 (50.0)	11 (78.6) 7 (50.0) 14 (100) 14 (100) 6 (42.9)	11 (68.8) 9 (56.3) 16 (100) 16 (100) 9 (56.3)	0.54^b^ 0.73^b^ NA^b^ NA^b^ 0.46^b^
Biochemical parameters fT4, pmol/l TSH, µU/ml Vitamin D, ng/ml Calcium, mmol/l Hemoglobin, g/dl Ferrum, mg/dl	15.5 (13.8-18.0) 1.63 (0.27-2.80) 40.0 (30.5-48.6) 2.43 (2.19-2.56) 14.1 (12.2-16.3) 93.5 46.0-145	15.9 (14.2-18.0) 1.70 (1.01-2.80) 40.0 (30.5-48.6) 2.41 (2.19-2.51) 14.2 (13.1-15.1) 99.5 (74.0-145)	15.1 (13.8-18.0) 1.57 (0.27-2.70) 39.5 (31.0-48.6) 2.44 (2.32-2.56) 13.8 (12.2-16.3) 89.5 (46.0-137)	0.11^a^ 0.58^a^ 0.67^a^ 0.22^a^ 0.06^a^ 0.58^a^

Data are presented as median (range min-max) or number (percentage).

a The Mann–Whitney test for comparison between groups.

b The Chi-square test for comparison between groups.

c Fisher exact test for comparison between groups.

d Antidepressants or antipsychotic drugs.

BMI, Body Mass Index; fT4, free thyroxine; IFG, impaired fasting glucose; HPAI, hypothalamic-pituitary-adrenal axis impairment; H,RT, hormone replacement therapy (estradiol and progesterone in female or testosterone in male), NA, not applicable; TSH, thyroid stimulating hormone; UPD15, uniparental maternal disomy of chromosome 15.

### Study protocol

The study protocol was approved by the Medical University of Warsaw Bioethics Committee (AKBE/88/2020). Written informed consent was obtained from the patients and their caregivers. The study was registered on the ClinicalTrials.gov (Identifier Number: NCT04700644).

Adrenal function was assessed using the high-dose short Synacthen test (HDSST) after the transition from pediatric to adult care (3) (VISIT I). Clinical assessments were then performed at the 6-month and 12-month follow-up visits (VISIT II and III, respectively).

At VISIT I, the adrenal function was assessed by:

- Clinical examination - in all patients we assessed symptoms and signs suggestive of adrenal insufficiency (including: fatigue, loss of appetite, muscle weakness, myalgia, arthralgia, weight loss > 3 kg, nausea, vomiting, abdominal pain, hypotension, hypoglycemia, hyponatremia) (16, 19).
- Measuring morning blood sample for cortisol, ACTH and DHEA-S.
- HDSST (*high-dose short synacthen test*) which is considered safe, reliable and convenient for testing the cortisol reserve (15, 16). The subjects were evaluated after a 12 hour overnight fast. The HDSST was performed at a similar time in all cases (between 8:00–10:00 in the morning). Blood samples for cortisol were measured at time points 0, 30 and 60 min after injecting 250 μg tetracosactide (1–24adrenocorticotropic hormone – ACTH) (Synacthen, Novartis, Naples, Italy).

HPA axis impairment (HPAI) was suspected in patients:

- With at least one clinical symptom suggestive of adrenal insufficiency and
- With a cortisol cut-off point of 500 nmol/L (<18.1 μg/dL) at 30 minutes, which was previously described as diagnostic of adrenal insufficiency in patients with PWS (2, 13),

In cases of suspected HPAI, all therapeutic options (close monitoring, hydrocortisone (HCT) stress-dose and low dose HCT treatment) were thoroughly discussed with patients and their caregivers. Hydrocortisone supplementation was initiated in dose of 15mg/m^2^, resulting in 10mg/day in two divided doses in the HPAI group (19, 20). During the follow-up, patients were asked to report possible side-effects of HCT therapy, including: infections, weight gain, increase in the amount of adipose tissue and metabolic abnormalities (impaired fasting glucose and lipid disorders).

None of the patients were on GLP-1 receptor agonists at the time of the study, although some were taking metformin.

### Hormone assays

All blood samples were immediately delivered to the laboratory and assayed. Blood samples for ACTH measurement were placed on ice until assayed.

All hormone assays were analyzed in the Central Laboratory of the Central Clinical Hospital of the University Clinical Centre at the Medical University of Warsaw. Cortisol levels were measured by electrochemiluminescence ECLIA method using Elecsys Cortisol II Cobas^®^ diagnostic kits (Roche Diagnostics Mannheim, Germany). The immunoassay for the *in vitro* quantitative determination of cortisol in human serum or plasma has a measuring range of 1.5–1750 nmol/L (0.054-63.4 μg/dL). Reference values for morning cortisol were: 133–537 nmol/L (4.82-19.5 μg/dL). DHEA-S concentration was measured by the electrochemiluminescence ECLIA method using Elecsys DHEA-S Cobas^®^ diagnostic kits (Roche Diagnostics Mannheim, Germany). The assay for the *in vitro* quantitative determination of DHEA-S in human serum or plasma has a measuring range of 0.003-27 μmol/L (0.010–100 ng/mL). Reference values were 5.72–13.35 μmol/L (211-492 μg/dL). ACTH was determined by electrochemiluminescence ECLIA method using Elecsys ACTH Cobas^®^ diagnostic kits (Roche Diagnostics Mannheim, Germany). Reference range for baseline ACTH was 1.6–13.9 pmol/L (7.22–63.3 pg/mL).

### Statistical analysis

Values are presented as median with ranges for continuous variables, and as a number of patients and percentage of sample for categorical variables. To assess the associations between variables the Mann–Whitney U test was used for continuous variables. The chi-square and the Fisher exact tests were used for categorical variables. Correlation was tested with Spearman’s method. The level of significance was set at p<0.05. The dependent groups were compared using Wilcoxon mathed–pairs test for continuous variables. Statistical analyses were performed by STATISTICA software ver.13.1 (TIBCO Software, Palo Alto, California, United States of America).

## Results

### Baseline characteristics

There were no significant differences between the studied groups regarding anthropometric parameters (age, sex, weight, body mass index), genetic abnormalities, concomitant diseases and additional treatments – **Table 1**.

### Adrenal function in adult PWS patients at I VISIT

**Figure 1:**
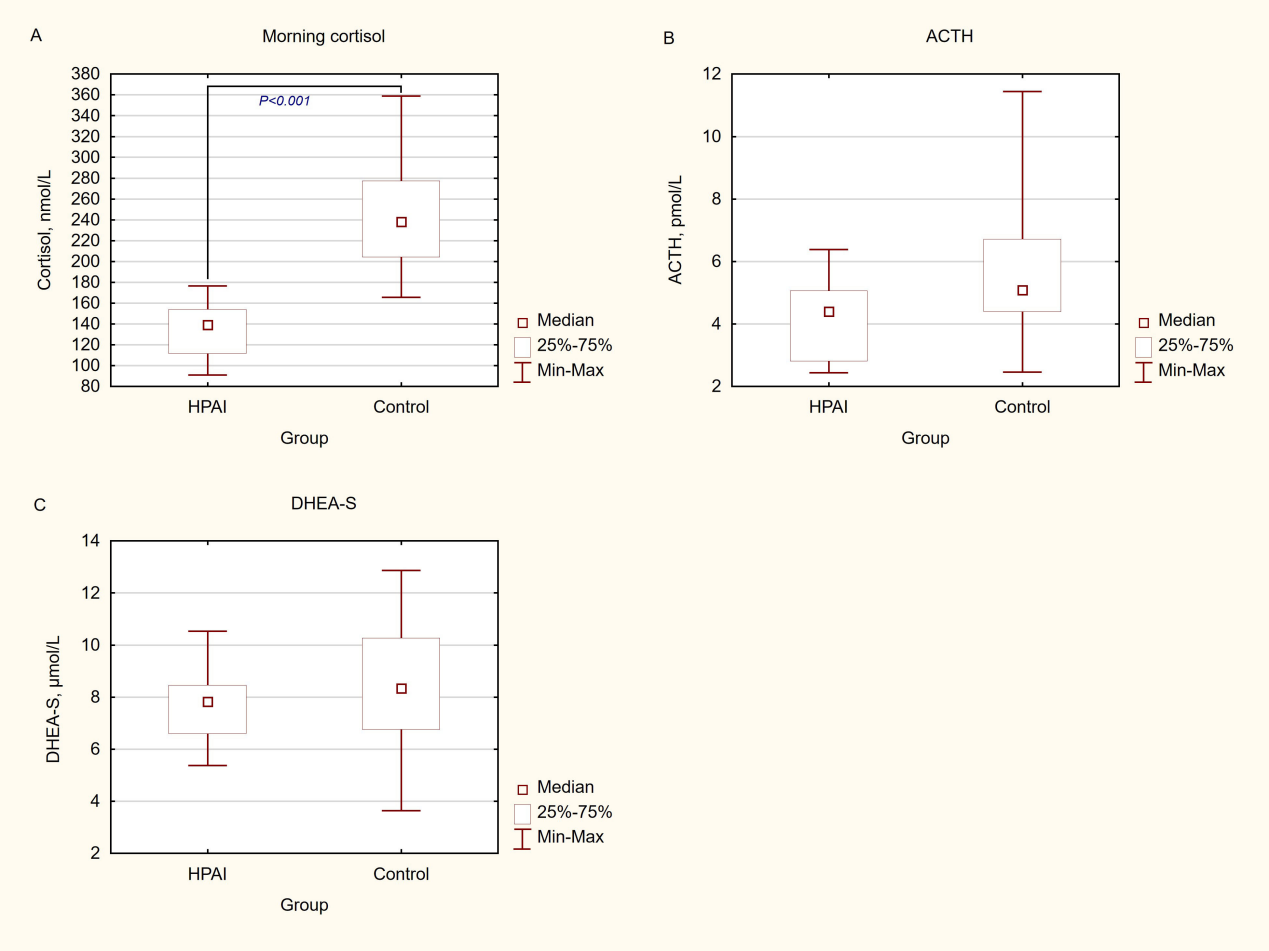
Morning cortisol, ACTH and DHEA-S values in patients with Prader-Willi Syndrome. **(A)** Morning cortisol. Reference values for morning cortisol are: 133–537 nmol/L (4.82-19.5 μg/dL). SI conversion factor: to convert cortisol from nmol/L to μg/dL divide by 27.59. **(B)** ACTH. Reference values for morning ACTH are: 1.6–13.9 pmol//L (7.22–63.3 pg/ml). SI conversion factor: to convert ACTH from pmol/L to pg/mL divide by 0.22. **(C)** DHEA-S. Reference values for DHEA-S in our study group are: 5.72–13.35 μmol/L (211-492 μg/dL). SI conversion factor: to convert DHEA-S from μmol/L to mg/dL multiply by 36.85. ACTH, adrenocorticotropine hormone; HPAI, hypothalamic-pituitary-adrenal axis impairment; DHEA-S, dehydroepiandrosterone sulfate..

The median (range) morning cortisol, ACTH and DHEA-S levels of the HPAI group were  173.9 nmol/L (91.0-359), 4.71 pmol/L (2.44-11.4) and 8.16 μmol/L (3.64-12.9), respectively. Cortisol levels below 133 nmol/L were found in 6 (20.0%) of cases. None of the patients had ACTH levels below 1.60 pmol/L. There were significant differences in morning (baseline) cortisol values between the HPAI and the Control group [median (range), 139 nmol/L (91.0-177), 238 nmol/L (166-359), respectively)] (*P*<0.001 (**Figure 1A**). There were no significant differences in the ACTH levels between the HPAI and the Control group [median (range), 4.40 pmol/L (2.44-6.38) and 5.08 pmol/L (2.46-11.4), respectively)] (*P*=0.06) (**Figure 1B**). Basal DHEA-S also did not differ between the two groups [median (range), 7,9 umol/L (5,2-10,4) and 8,3 (3,8-12,9), respectively)] (*P=*0.37) (**Figure 1C**). There were no significant differences in hormonal levels between the patients with UPD and patients with 15q (11-13) deletion and between two sexes.

**Figure 2:**
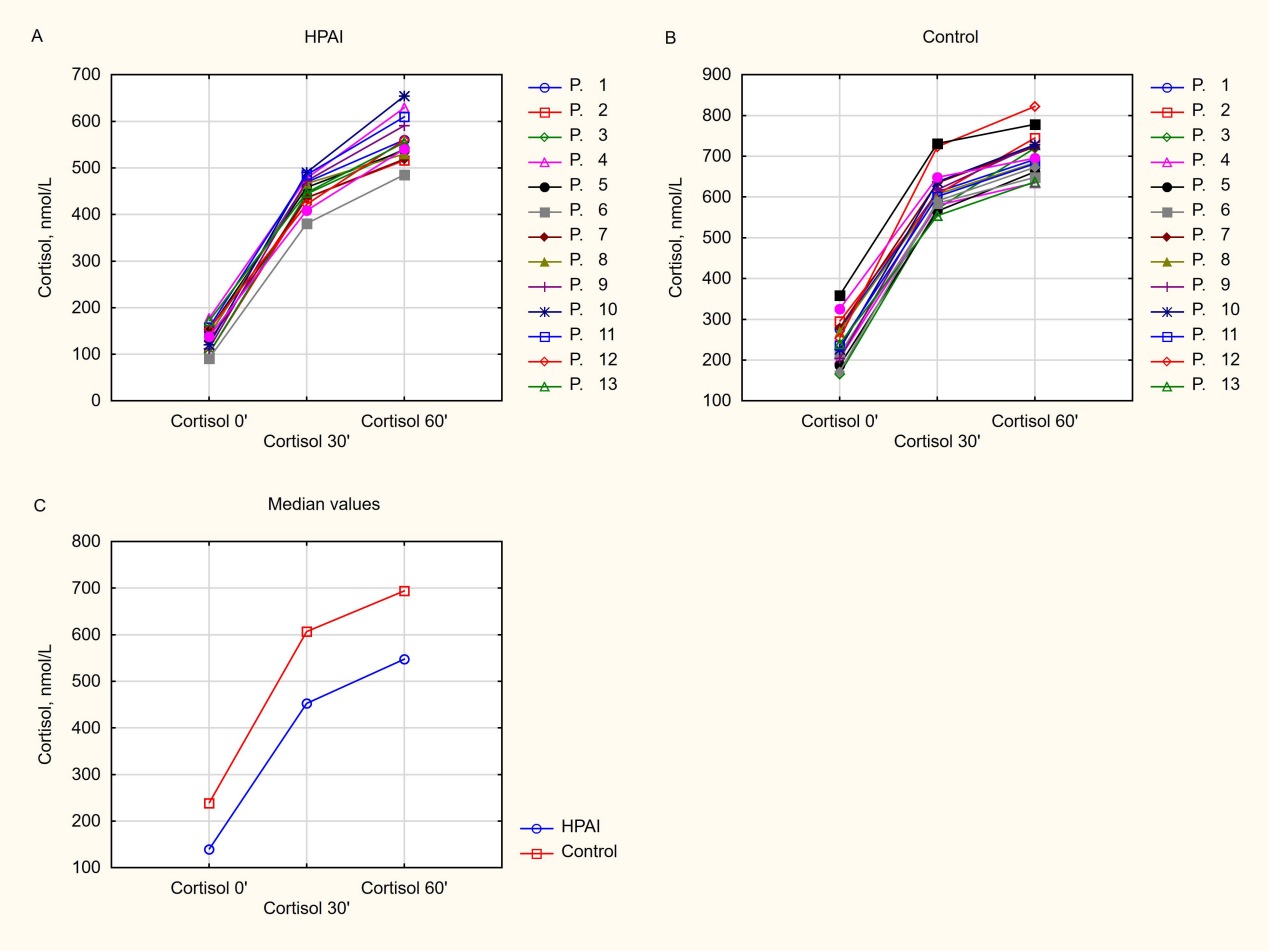
High-dose short synacthen test results in the study groups. **(A)** Hypothalamic-pituitary-adrenal axis impairment (HPAI) group **(B)** Control group. **(C)** Median values in the study groups. Morning (baseline) cortisol and peak cortisol levels of the HDSST at 30’ and 60’ are shown. Reference values for morning cortisol are: 133–537 nmol/L (4.82-19.5 μg/dL). SI conversion factor: to convert cortisol from nmol/L to μg/dL divide by 27.59. HPAI, hypothalamic-pituitary-adrenal axis impairment.

Results of the HDSST are shown in **Figure 2**. Fourteen of the 30 patients (46.7%) showed a 30-min cortisol peak <500 nmol/L, and were assigned to the HPAI group. Five of these patients had a 30-min cortisol response below 440 nmol/L and 6 had basal cortisol levels below 133 nmol/L. Peak cortisol levels at 30’ were significantly lower in the HPAI compared to the Control group, the median (range) being: 452 nmol/L (381-532) and 607 nmol/L (555-731), respectively (*P*<0.001). All patients but one (in the HPAI group) showed a 60-min cortisol peak to HDSST ≥500 nmol/L. However, peak cortisol levels at 60’ were also significantly lower in the HPAI group when compared with the Control group, the median (range) being: 548 nmol/L (486-654) and 694 nmol/L (635-822), respectively (*P*<0.001). Also Δ cortisol 30’ was significantly lower in the HPAI compared to the Control group. Median Δ cortisol 30’ was 317 nmol/L and 371 nmol/L, respectively (*P*=0.002). But there was no significant difference regarding Δ cortisol 60’ in the HPAI and the Control group with median 410 nmol/L and 445 nmol/L, respectively (*P*=0.07). Correlation analysis revealed that basal cortisol was positively correlated with cortisol levels at both 30’ and 60’ of the HDSST (r = 0.872, *P* < 0.001 and r = 0.829, *P* < 0.001, respectively).

Signs and symptoms suggestive of hypothalamic-pituitary-adrenal axis impairment in the study group at I VISIT are presented in **Table 2**. Patients in the HPAI group reported muscle weakness and myalgia significantly more often than the Control group (**Table 2**). None of the patients had been previously hospitalized due to an adrenal crisis or other life-threatening conditions.

**Table 2:** Signs and symptoms of adrenal insufficiency in adult patients with Prader-Willi syndrome at I Visit.

Symptoms/signs	HPAI group (n=14)	Control group (n=16)	*P* value ^a^
Fatigue	13 (92.9)	10 (62.5)	0.09
Loss of appetite	0	0	–
Muscle weakness	12 (85.7)	4 (25.0)	0.001
Myalgia	5 (35.7)	0	0.01
Arthralgia	1 (7.14)	0	0.47
Weight loss	0	0	–
Nausea	0	0	–
Vomiting	0	0	–
Abdominal pain	0	0	–
Hypotension	0	0	–
Hypoglycemia	0	0	–
Hyponatremia	0	0	–

Data are presented as number (percentage).

a Fisher exact test for comparison between groups

HPAI, hypothalamo-pituitary-adrenal axis impairment

All patients with HPA axis impairment agreed to receive HCT supplementation resulting in a daily dose of 10 mg/day, taken in two divided doses. After 6 and 12 months of HCT therapy patients in the HPAI group improved significantly in terms of fatigue, myalgia and muscle weakness, which was confirmed by the observation of their caregivers (**Table 3**). After 12 months all but one patient decided to continue HCT supplementation at a lower dose (5 mg per day). No significant adverse effects of HCT treatment were observed within the 6 and 12 months of observation. Body weight and BMI measurements evaluated at 6 and 12 months of treatment showed no significant differences from baseline. Moreover, the percentage of body fat decreased significantly after 6, and 12 months of the treatment (**Table 3**). A similar trend in weight and fat tissue was observed in both rhGH treated and not treated patients.

**Table 3:** The effects of hydrocortisone supplementation therapy in the study groups.

Variable	VISIT I Before treatment	VISIT II After 6 months	VISIT III After 12 months	*P* value: Visit II vs. I	*P* value: Visit III vs. I
HPAI group (n=14)					
Fatigue	13 (92.9)	8 (57.1)	1 (7.14)	0.08^b^	<0.001^b^
Muscle weakness	12 (85.7)	7 (50.0)	2 (14.3)	0.10^b^	<0.001^b^
Myalgia	5 (35.7)	2 (14.3)	1 (7.14)	0.40^b^	0.16^b^
IFG	7 (50.0)	7 (50.0)	7 (50.0)	1.00^c^	1.00^c^
Weight, kg	68.0 (42.0-120)	69.9 (43.0-122)	65.6 (43.0-120)	0.36^a^	0.67^a^
% body Fat	23.8 (16.0-64.0)	19.1 (14.0-60.0)	16.5 (10.0-52.0)	0.003^a^	0.003^a^
Control group (n=16)					
Fatigue	10 (62.5)	6 (37.5)	4 (25.0)	0.16^c^	0.07^b^
Muscle Weakness	4 (25.0)	3 (18.8)	2 (12.5)	0.70^b^	0.66^b^
Myalgia	0	0	0	NA^b^	NA^b^
IFG	7 (43.8)	7 (43.8)	7 (43.8)	1.00^c^	1.00^c^
Weight, kg	77.0 (48.0-168)	80.8 (48.0-165)	79.0 (48.5-164)	0.73^a^	0.78^a^
% body Fat	33.4 (12.8-55.0)	29.1 (12.0-51.0)	29.3 (13.0-51.0)	0.16^a^	0.06^a^

Data are presented as median (range min-max) or number (percentage).

a the Wilcoxon matched-pairs test for comparison between variables.

b Fisher exact test for comparison between groups.

c The Chi-square test for comparison between groups.

HPAI, hypothalamo-pituitary-adrenal axis impairment; IFG, impaired fasting glucose, NA, not applicable.

Similar to the HPAI group, after 6 and 12 months of observation patients in the Control group improved in terms of fatigue, myalgia and muscle weakness (**Table 3**). Body weight and BMI measurements evaluated at 6 and 12 months of observation showed no significant differences from baseline. The percentage of body fat did not differ significantly after 6 and 12 months of the observation (**Table 3**). A decrease in body fat percentage was observed in some subjects, possibly due to the increase in muscle strength and physical activity (reported by caregivers). Increased daily physical activity was noted in all participants.

Before starting the HCT treatment, 7 patients with HPAI had IFG treated with metformin, which did not get worse after 6, and 12 months of the HCT treatment. None of the other patients developed new carbohydrate disorders during 12 months of the HCT treatment.

## Discussion

The diagnosis of HPA axis impairment in patients with PWS remains challenging, because the clinical presentation in this population may be vague, as syndromal characteristics may overlap with adrenal insufficiency symptoms. The age of patients in a given study cohort may also have a significant impact on the results. Moreover, the interpretation of laboratory tests depends on the test used, as well as the assumed cut-off points, which makes comparisons between different study designs difficult. Although ITT is considered the ‘gold standard’ for the assessment of central adrenal insufficiency, this test carries inherent risks, including severe hypoglycemia and (like the metyrapone test); it also requires hospitalization. Thus it poses an additional stress for PWS patients, which may have a negative effect on their mental state, exacerbate anxiety disorders, cause aggressive behavior, leading to false test results (21–24). Therefore, in our study we decided to employ HDSST, which is an easy, fast, safe, reliable and convenient method for testing cortisol reserve (2, 14, 24, 25).

However, as mentioned above, the interpretation of the HDSST in patients with suspected HPA impairment is not straightforward, and the prevalence largely depends on the adopted cut-off points. To illustrate the significance of this point let consider the following. We found, that fourteen of the 30 patients (46.7%) showed a 30-min cortisol peak <500 nmol/L, and were assigned to the HPAI group. Adopting 600 nmol/l cut-off at 30 min would translate into a diagnosis of HPAI in 76.2% of our patients (n=22/30) (15). Endocrine Society Guidelines suggest to use HDSST to diagnose both primary and secondary adrenal insufficiency: peak cortisol levels < 500 nmol/L at 30 or 60 min indicates adrenal insufficiency. In our study, all patients but one had a delayed peak response, without evidence of adrenal atrophy, at 500 nmol/l cut-off point (26). A similarly delayed response was observed by Oto et al. (27).

The results of our study are in agreement with previous studies using HDSST (cut-off 500 nmol at 30 min), suggesting a high level of prevalence of HPA disfunction in adult patients with PWS (15).

Adrenal atrophy due to secondary or tertiary AI of a certain duration, as well as clinically significant CAI is usually revealed by HDSST (21, 22, 25). A similar prevalence of HPAI among adults with PWS was observed in the de Lind van Wijngaarden study (2008), where a metyrapon test was used to evaluate the condition (8). In two studies, where 1ug tetrazacotide test was performed (LDSST), adrenal insufficiency was diagnosed in 14.3% and 7.5% of cases, respectively (2, 14). These differences may be largely likely attributed to the age of the study participants (14, 28). This is consistent with previous studies, where percentage of adrenal insufficiency cases in adults (7.5%) was found to be higher than in childhood (4.8%) (2, 12–14), indicating that the risk of HPAI increases with age. On the other hand, in a large multicenter study, Rosenberg et al. found that central adrenal insufficiency is rare (1.2%) in adults with PWS using a ITT (cut off > 450–500 nmol/l at 30, 60 or 90 min) or metyrapone test (11-deoxycortisol >230 nmol/L was considered sufficient) (29). It should also be stressed that normal body weight was observed in most patients participating in our study, which is also an important differentiating element from other research to date.

We observed that morning cortisol values differed between patients with HPA axis impairment and Control group. A positive correlation with basal cortisol and HDSSD was found at both 30 and 60 minutes (**Table 3**), in concord with results obtained in previous studies (2). Thus we propose that an assessment of basal cortisol could be used as a HPA impairment screening test in patients with PWS.

Importantly, in our study, we observed that PWS patients with HPAI more often report fatigue, muscle weakness, muscle and joint pain compared to the Control group.

Similarly to previous reports, we did not observe other symptoms and signs associated with adrenal insufficiency in this specific population (29).

To the best of our knowledge this is the first study showing the effects of HCT therapy in symptomatic PWS patients with HPAI, suggesting a possible positive effect of such an intervention on muscle weakness and fatigue (30). Such effect was confirmed by the caregivers. Following the 12 month intervention all but one patient supported by caregivers decided to continue HCT supplementation at a lower dose (ranging from 5 to 10 mg per day). What is also very important, we did not observe any metabolic complications or weight gain. On the contrary, a decrease in body fat percentage was observed in some subjects, possibly due to the increase in muscle strength and physical activity (reported by caregivers). Increased daily physical activity was noted in all participants, which is crucial, as this factor may by itself positively influence health-related quality of life in youth with obesity and PWS (31, 32).

Our study has several important limitations: (i) the study was retrospective; (ii) the study group was relatively small; (iii) only one test was used to diagnose HPA axis; (iv) all patients with HPAI received treatment, and (v) the 1 year follow-up does not exclude important late adverse effects of HCT treatment.

It is worth noting that interpretation of the clinical presentation and endocrine test results in PWS has previously proved puzzling, as attested by the initial controversies surrounding growth hormone deficiency diagnosis and treatment in this condition (33).

In conclusion, the results of our study suggest that the hypothalamic-pituitary-adrenal axis should be routinely evaluated in adult patients with PWS. However, it is yet to be established which screening and confirmatory tests are the best in this case. Our result support the notion that morning cortisol levels and HDSST may be useful in diagnosing HPAI in this population, however the best cut-off values to rule-in or rule-out the diagnosis in PWS remain unknown. For the first time we have shown that short term, low-dose HCT treatment in patients with PWS and HPAI is safe and can reduce symptoms of fatigue and muscle weakness (34). However, the benefits and adverse effects of HCT treatment require confirmation in prospective, placebo-controlled randomized clinical studies.

## Data Availability

The raw data supporting the conclusions of this article will be made available by the authors, without undue reservation.
